# Plug-assisted retrograde transvenous obliteration for the treatment of gastric variceal bleeding in absence of gastrorenal or gastrocaval shunt: A case report

**DOI:** 10.1097/MD.0000000000032013

**Published:** 2022-12-09

**Authors:** Jongjoon Shim, Jae Myeong Lee, Youngjong Cho

**Affiliations:** a Department of Radiology, University of Soonchunhyang College of Medicine, Soonchunhyang University Bucheon Hospital, Bucheon-si, Gyeonggi-do, Korea; b Department of Radiology, University of Ulsan College of Medicine, Gangneung Asan Hospital, Gangneung, Gangwon-do, Korea.

**Keywords:** gastric varix, pericardial vein, plug-assisted retrograde transvenous obliteration

## Abstract

Plug-assisted retrograde tansvenous obliteration (PARTO) is commonly used to treat gastric variceal bleeding. PARTO is typically performed via the gastrorenal shunt or gastrocaval shunt and there have been no reports of PARTO performed through the pericardial vein in cases of gastric varices in which there is no gastrorenal or gastrocaval shunt. Herein, we report a case of PARTO was performed to treat gastric variceal bleeding in a patient with a gastric varix without a gastrorenal or gastrocaval shunt.

**Patient concerns:**

A 54-year-old man with alcoholic liver cirrhosis presented to the emergency room with hematemesis and melena. At presentation, the patient’s blood pressure was 130/70 mm Hg and hemoglobin level was 10.1 g/dL.

**Diagnoses:**

Computed tomography (CT) scan and endoscopic examination revealed a gastric varix at the gastric fundus.

**Interventions:**

PARTO was performed to treatment of gastric variceal bleeding via the pericardial vein.

**Outcomes:**

The patient did not show any signs of variceal bleeding after the procedure, and follow-up CT at 3 weeks showed complete resolution of the gastric varix.

**Lessons:**

Although PARTO is technically difficult to perform through pathways other than the gastrorenal or gastrocaval shunt, it can be a beneficial alternative in cases in which other treatments fail or are not feasible.

## 1. Introduction

Gastric varices occur in 20% to 30% of patients with portal hypertension due to liver cirrhosis.^[1,2]^ Although the frequency of gastric variceal bleeding is lower than that of esophageal variceal bleeding, the former has a higher mortality rate because of the large amount of bleeding and high rebleeding rate.^[2–4]^ Most of the gastric varices drain into the left renal vein via a gastrorenal shunt.^[1]^ Based on this concept, the procedure of balloon-occluded retrograde transvenous obliteration (BRTO) was developed for the management of gastric varices, wherein the gastrorenal shunt is occluded with a balloon and retrograde injection of a sclerosing agent into the gastric varix.^[5]^ Since its inception, BRTO has been widely applied to the treatment of gastric varices.

Recently, Gwon et al introduced the plug-assisted retrograde transvenous obliteration (PARTO) technique, which uses a vascular plug and retrograde injection of gelatin sponge particles to induce thrombosis of the gastric varix and gastrorenal/gastrocaval shunt.^[6,7]^ This method has gained popularity as an alternative treatment method for gastric varices.

To the best of our knowledge, there have been no reports of PARTO performed through the pericardial vein in cases of gastric varix without the presence of a gastrorenal or gastrocaval shunt. Herein, we report the first case of gastric varix management using PARTO through the pericardial vein in a patient without gastrorenal or gastrocaval shunt.

## 2. Case report

A 54-year-old man presented to the emergency room with hematemesis and melena. He was diagnosed with alcoholic liver cirrhosis, but did not receive any specific treatment. The patient’s blood pressure was 130/70 mm Hg and hemoglobin level was 10.1 g/dL.

**Figure 1.**
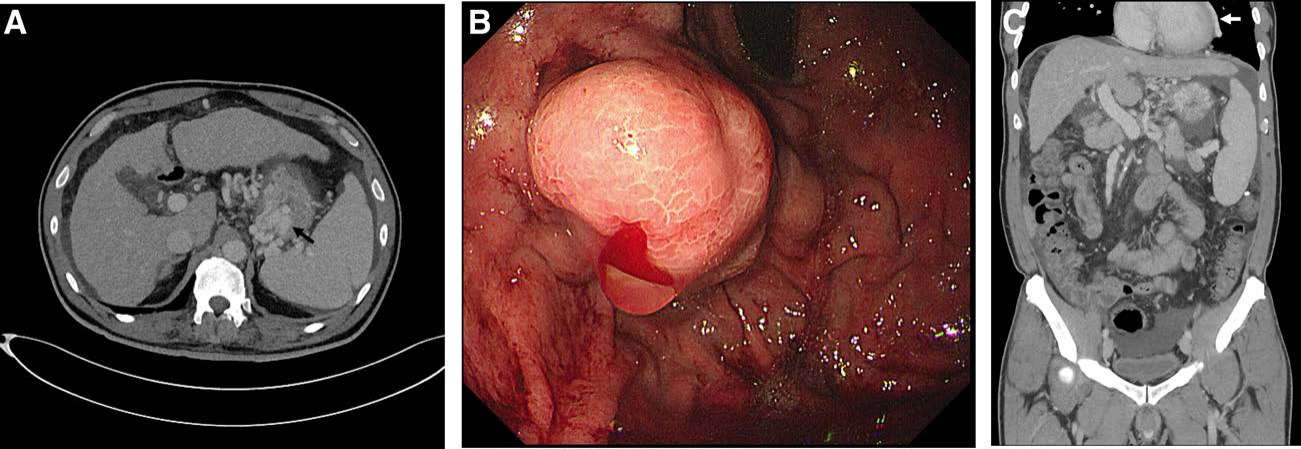
(A) CT image showing a tortuous and enlarged gastric varix (arrow) in the gastric fundus. (B) Endoscopic image of the gastric varix with bleeding appearing as a polypoid submucosal mass. (C) Coronal CT image of the pericardial vein (white arrow) running along the left border of the heart. CT = computed tomography.

Computed tomography (CT) revealed a large and tortuous varix at the gastric fundus; however, no contrast medium extravasation was noted in the gastrointestinal tract lumen (Fig. 1A). This was followed by endoscopic examination that revealed the gastric varix to be a polypoid submucosal mass with bleeding (Fig. 1B). Thereafter, an endoscopic histoacryl injection was administered to the bleeding gastric varix to achieve hemostasis.

**Figure 2.**
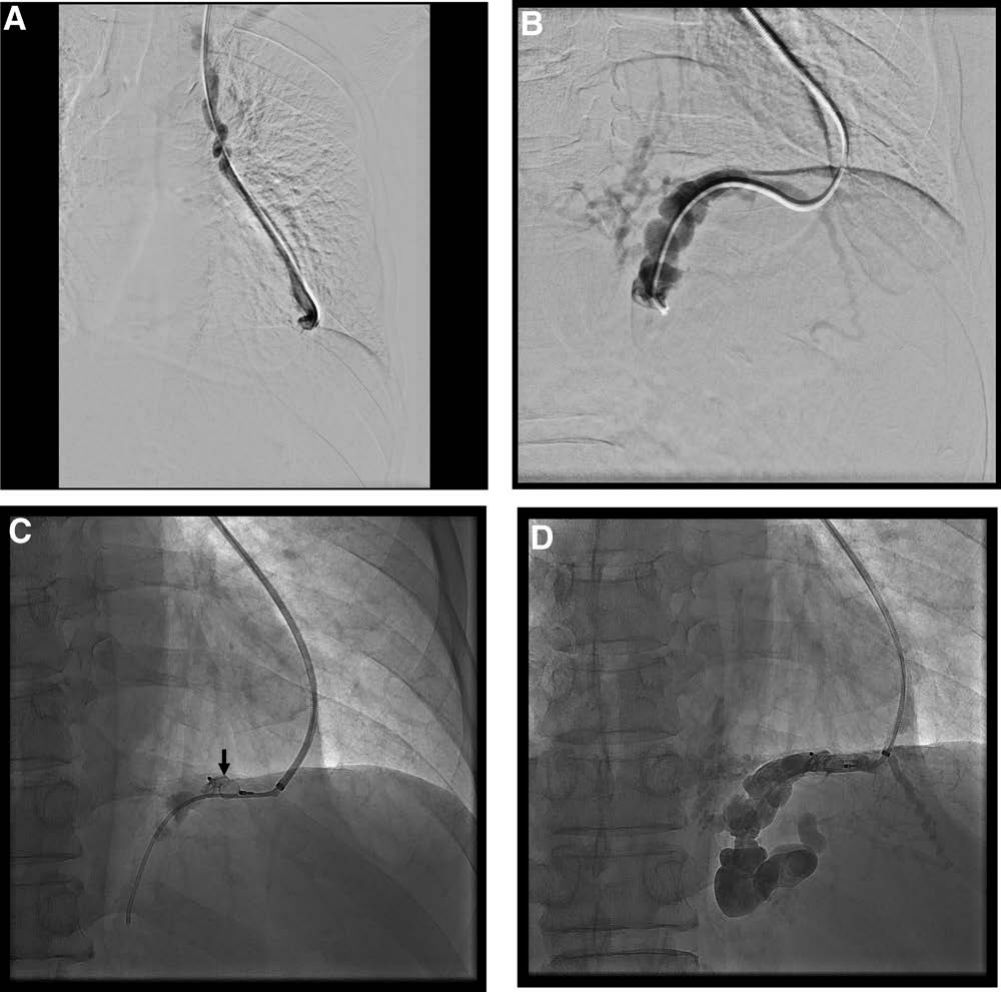
(A) Venography shows the pericardial vein draining into the left brachiocephalic vein. (B) Venography via 4Fr. catheter showing the ascending portion of the left inferior phrenic vein. It serves as an efferent vein of the gastric varix. (C) 8 mm vascular plug (arrow) is placed in the transverse portion of the left inferior phrenic vein. (D) Fluoroscopy images showing an opacified gastric varix filled with a mixture of gelatin sponge particles and a contrast agent.

The day after endoscopic hemostasis, melena persist, the patient’s hemoglobin level dropped to 8.5 g/dL despite transfusion, and his blood pressure was 100/60 mm Hg. Therefore, it was determined that the gastric variceal bleeding had not stopped. The CT examination showed no gastrorenal or gastrocaval shunt, but thickening of the pericardial vein connected to the left inferior phrenic vein (Fig. 2A). As no alternative treatment was feasible in this scenario, we decided to perform PARTO through the pericardial vein.

The left internal jugular vein was punctured under US guidance. 7Fr. guiding sheath (Flexor Raabe guiding sheath, COOK, Bloomington, IN) was introduced into the left brachiocephalic vein via the left internal jugular vein. Thereafter, catheterization of the pericardial vein was attempted using a 4Fr. angiographic catheter (Berenstein, Terumo, Tokyo, Japan) and 0.035 inch hydrophilic guide wire (Terumo, Tokyo, Japan). Venography was performed after the successful insertion of the angiographic catheter into the pericardial vein (Fig. 2B). The efferent vein of the gastric varix was observed on venography after a 4Fr. angiographic catheter and 0.035 guide wire were advanced into the left inferior phrenic vein. Next, a 7Fr. guiding sheath was advanced into the left inferior phrenic vein along the angiographic catheter and guide wire, and a 10mm vascular plug (Amplazer vascular plug type II, Abbott, Plymouth, MN) was placed into the vein through the guiding sheath (Fig. 2C). Subsquently, a 4Fr. angiographic catheter was introduced into the proximal portion of the left inferior phrenic vein, and gelatin sponge particles were injected through the catheter until the gastric varix was opacified (Fig. 2D).

**Figure 3.**
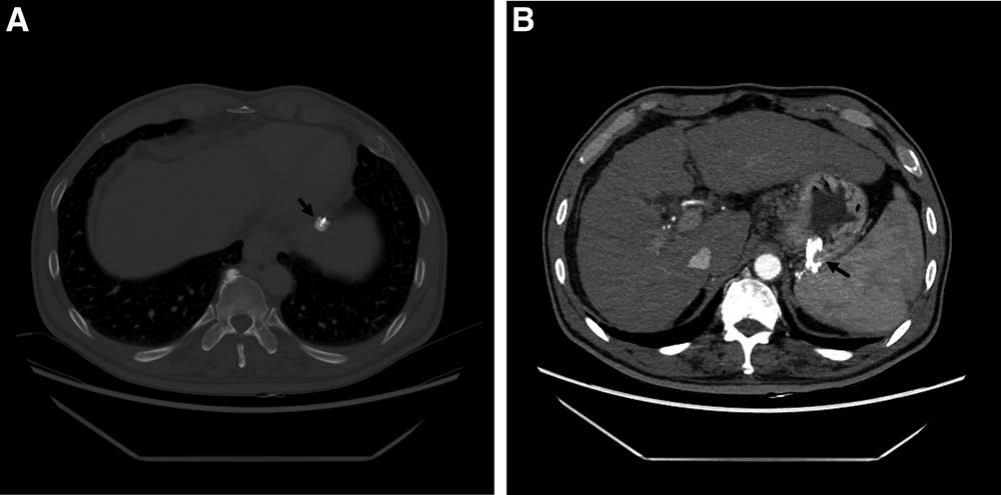
(A) Vascular plug (arrow) is placed in the embolized transverse portion of left inferior phrenic vein. (B) Follow-up CT image showing complete disappearance of the gastric varix. Histoacryl (arrow) injected endoscopically can also be observed. CT = computed tomography.

The day after PARTO, there were no signs of bleeding such as melena or hematemesis. And hemoglobin level was elevated to 11.3 g/dL. The patient was discharged 6 days after PARTO. A follow-up CT-scan was conducted 3 weeks after PARTO, which confirmed that the vascular plug in the left inferior phrenic vein was located normally (Fig. 3A), and that the gastric varix had disappeared (Fig. 3B).

## 3. Discussion

Endoscopic treatments such as injection sclerotherapy and variceal ligation are effective for esophageal varices but less effective for gastric varices because of the faster and greater volume of gastric variceal blood flow.^[1]^ Therefore, gastric varices are primarily managed via endovascular treatments such as PARTO or BRTO.

Ethanolamine oleate (EO) is the most commonly used sclerosing agent in BRTO; however, BRTO using EO can lead to pulmonary edema, disseminated intravascular coagulation, anaphylactic reaction and severe renal dysfunction.^[8]^ Although these complications were resolved by replacing EO with 3% sodium tetradecyl sulfate, the disadvantage of maintaining ballooning for an extended period for vascular sclerosis remained.^[9]^

Compared to BRTO, PARTO has several advantages. The procedure time for PARTO is shorter than that for BRTO. In addition, PARTO does not require balloon indwelling, which reduces patient discomfort.^[6]^ Therefore, recently, PARTO has gained greater recognition than BRTO in the treatment of gastric varices.

Gastric varices formed from afferent veins such as the left gastric vein, posterior gastric vein, and short gastric vein can form connections with systemic veins through various pathways. 80% to 85% of gastric varices drain into the left renal vein via the gastrorenal shunt. In cases of gastric varices without a gastrorenal shunt, several other pathways may serve as the primary draining route. One such pathway, known as the gastrocaval shunt, involves the transverse part of the inferior phrenic vein, which runs under the diaphragm and connects the varix directly to the inferior vena cava. Another route of drainage of the gastric varix is via the pericardial vein into the left brachiocephalic vein.^[10–13]^

PARTO and BRTO are methods of gastric varix management that use a retrograde approach via the draining vein connected to the systemic vein. Jang et al^[7]^ described a case in which PARTO was performed through a gastrocaval shunt in a patient who had gastric variceal bleeding without a gastrorenal shunt. Furthermore, in a study by Araki et al,^[14]^ BRTO was performed via other pathways in patients with gastric varices without gastrorenal shunts. However, the present case report is the first to describe PARTO performed through the pericardial vein in a patient with gastric varix without a gastrorenal or gastrocaval shunt.

There are several technical difficulties associated with performing PARTO via the pericardial vein. First, it is difficult to identify the point where the pericardial vein drains into the left brachiocephalic vein. Second, the effect of the vascular plug on the heart cannot be predicted when it is placed in the pericardial vein attached to the heart. Therefore, it is necessary to place the vascular plug in the left inferior phrenic vein connected to the gastric varix; for this, the guiding sheath must be correctly inserted into the left inferior phrenic vein. However, the pathway between the pericardial vein and left inferior phrenic vein forms an acute angle, making advancement of the guiding sheath difficult. In addition, the small diameter of the pericardial and left inferior phrenic veins is another factor that makes device advancement in these veins difficult.

Despite these difficulties, PARTO through other pathways, such as the pericardial vein, can be attempted when there is no gastrorenal or gastrocaval shunts and other treatments are ineffective. Therefore, it is necessary to recognize the presence of pathways other than the gastrorenal shunt or gastrocaval shunt through CT evaluation of the gastric varix before performing PARTO.

## Author contributions

**Conceptualization:** Jongjoon Shim.

**Funding acquisition:** Jongjoon Shim.

**Resources:** Jongjoon Shim.

**Supervision:** Jae Myeong Lee.

**Writing – original draft:** Jongjoon Shim.

**Writing – review & editing:** Jae Myeong Lee, Youngjong Cho.
